# Healthcare Disparities in the Treatment and Outcomes of Hepatocellular Carcinoma in South Africa

**DOI:** 10.1002/wjs.12559

**Published:** 2025-03-18

**Authors:** Sanju Sobnach, Muhammad Emmamally, Keith Venter, Jake Krige, Marc Bernon, Christo Kloppers, Mark W. Sonderup, C. Wendy Spearman, Rufaida Khan, Urda Kotze, Eduard Jonas

**Affiliations:** ^1^ Surgical Gastroenterology Unit Division of General Surgery Department of Surgery Faculty of Health Sciences University of Cape Town Cape Town South Africa; ^2^ Division of Hepatology Department of Medicine Faculty of Health Sciences University of Cape Town Cape Town South Africa

**Keywords:** disparities, hepatocellular carcinoma, outcomes, private healthcare, South Africa

## Abstract

**Background:**

The impact of healthcare disparities on the outcomes for hepatocellular carcinoma (HCC) has not been explored in South Africa. This study aims to evaluate and compare the presentation, treatment, and outcomes of HCC in a cohort of patients treated in the public and private sectors.

**Methods:**

The records of 551 consecutive patients treated at a public hospital compared to those of 51 treated in the private sector from 1 December 2001 to 29 February 2024 were retrospectively reviewed.

**Results:**

Patients managed in the public sector were significantly younger (mean age: 49.6 ± 14.8 vs. 59.6 ± 14.3 years, *p* < 0.00001) and more likely to have hepatitis B virus (HBV)‐related HCC (62.1% vs. 17.6%, *p* < 0.00001). The prevalence of multifocal disease (59.2% vs. 15.7%, *p* < 0.00001), portal vein tumor thrombosis (44.6% vs. 5.9%, *p* < 0.00001), and pulmonary metastases (16.2% vs. 3.9%, *p* = 0.0143) was significantly higher in public sector patients. A significantly higher number of public sector patients received best supportive care as their only treatment (69.7% vs. 15.7%, *p* < 0.00001). A higher proportion of private sector patients were treated with curative‐intended therapies (ablation, liver resection, and liver transplantation) and transarterial modalities. Median survival was lower in public sector patients (68 [IQR: 25–232] vs. 703 [IQR: 388–1327] days, *p* < 0.001). There was no difference in survival between public and private sector patients treated with curative‐intended therapies.

**Conclusions:**

In the public sector, patients present with more advanced HCC, which limits their access to curative‐intended therapies, resulting in lower survival. Patients treated with curative‐intended therapies have similar survival rates in the public and private sectors. With the introduction of universal health coverage through the National Health Insurance program in South Africa, these data highlight the gaps in HCC care in the public sector, where health initiatives such as HBV vaccination, early treatment of HBV, patient education, and screening of at‐risk patients should be prioritized.

## Graphical abstract

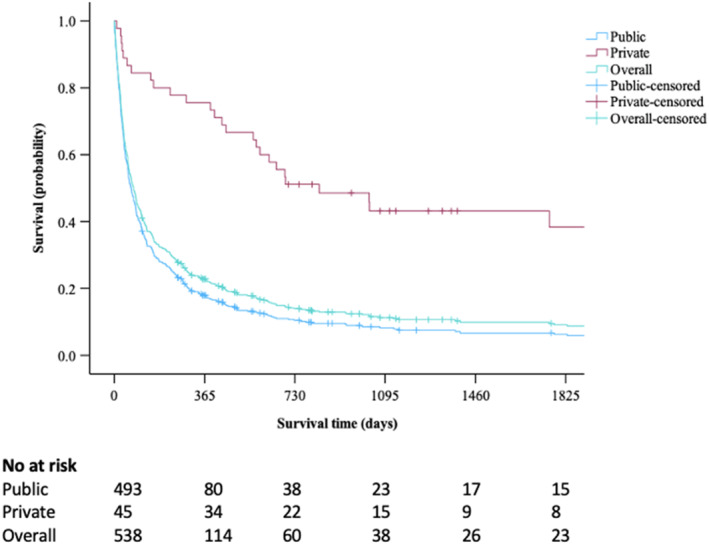

## 1 Introduction

South Africa has the highest wealth inequality globally, with 86% of the national wealth aggregate held by 10% of the population [1]. Over the last three decades, this wealth gap has widened significantly and translated partly into healthcare disparities in the clinical outcomes of local disease burden [2, 3, 4, 5, 6, 7, 8, 9, 10, 11]. Although the constitution guarantees access to healthcare for all, there is a complex public–private dichotomy in South Africa. Sixteen percent of the population have private healthcare insurance, whereas the remaining 84% are uninsured. The latter rely on out‐of‐pocket expenditure and public hospitals for medical care, which are often under‐resourced and overburdened [1, 2, 3, 4]. The impact of these healthcare disparities on cancer treatments and their respective outcomes has not been evaluated in detail in South Africa. This is in a context where the cancer burden in sub‐Saharan Africa (SSA) is expected to undergo an 85% increase, from 12.7 million new cases in 2008 to 22.2 million by 2030 [12].

Hepatocellular carcinoma (HCC) is a leading cause of cancer‐related mortality among young men and women in SSA. Most patients present with advanced disease, and less than 1% benefit from curative‐intended therapies. Data from high‐income countries (HICs) and low‐ and middle‐income countries (LMICs) have shown that privately insured HCC patients have a higher socioeconomic status [13, 14, 15, 16, 17, 18, 19]. As a result, they have better access to screening and curative‐intended therapies (ablation, liver resection, and liver transplantation), which ultimately results in improved survival [20, 21, 22, 23, 24, 25, 26, 27]. Establishing whether there are disparities in care and outcomes in highly prevalent cancers, such as HCC, in South Africa is a critical component in developing strategies aimed at directing healthcare resources to currently disadvantaged groups of the population.

Thus, in this study, we describe, compare, and analyze the demographics, presentation, treatment, and outcomes of HCC patients treated at Groote Schuur Hospital (GSH), the state‐funded tertiary‐level public sector hospital affiliated with the University of Cape Town, Cape Town, South Africa, and the University of Cape Town Private Academic Hospital (UCTPAH), a tertiary university‐affiliated private healthcare facility.

## 2 Methods

This study was approved by the Human Research Ethics Committee of the Faculty of Health Sciences at the University of Cape Town (IRB00001938, HREC: 391/2024). Patients with HCC treated at GSH and UCTPAH, Cape Town, South Africa, over a 23‐year period from 1 December 2001 to 29 February 2024 were included in this retrospective observational cohort study. Data were retrieved from two prospectively maintained HCC databases: a paper‐based database, which included HCC patients treated from 1 February 2001 to 31 December 2016 and a secure faculty‐managed REDCap (Research Electronic Data Capture) platform hosted by the University of Cape Town (IRB00001938, R003/2019), which captured patient data from 1 January 2017.

Patient demographics, Eastern Cooperative Oncology Group (ECOG) performance status (PS), symptoms and their duration, clinical examination findings, comorbidities, and lifestyle factors (alcohol consumption, smoking) were extracted. The following laboratory investigations were recorded: full blood count, international normalized ratio (INR), renal and liver function tests (LFTs), serum alpha‐fetoprotein (AFP), hepatitis B virus (HBV), hepatitis C virus (HCV), human immunodeficiency virus (HIV) serology, and tumor histology. Radiology reports (abdominal ultrasound (US), computed tomography (CT), and magnetic resonance imaging (MRI)) were evaluated, and relevant findings were recorded. The Child–Turcotte–Pugh (CTP) score, model for end‐stage liver disease‐sodium (MELD‐Na) score, and Barcelona Clinic Liver Cancer (BCLC) stage were computed and analyzed. Treatments and long‐term outcomes were recorded and analyzed across the two patient groups (GSH vs. UCTPAH).

Frequencies and percentages were used to present categorical variables, and comparisons were made using the chi‐square test or Fisher’s exact test. Continuous variables were expressed as means using the Student’s *t*‐test or the Wilcoxon rank sum test. Survival curves were generated using the Kaplan–Meier method and compared using the log‐rank test. Survival was expressed as a median with respective interquartile ranges (IQRs). Factors impacting survival were assessed using univariate and multivariate Cox proportional hazards regression models. Only variables with univariate significance were entered into a multivariate Cox model. Variables with the highest *p*‐values were removed from the model using backward stepwise elimination until all variables in the model remained statistically significant. Statistical significance was based on a two‐sided test at 5%.

## 3 Results

A total of 602 patients with HCC were included in the study, of whom 551 were treated in the public health GSH facility and 51 in the privately funded UCTPAH (Table 1). The sex distribution was similar in both cohorts, but patients treated in the public sector were, on average, 10 years younger, mean 49.6 ± 14.8 versus 59.6 ± 14.3 years (*p* < 0.00001). The BMI was lower in public sector patients (23.6 ± 5.6 vs. 27.6 ± 4.7 kg/m^2^, *p* = 0.00857) with an ECOG PS of 3 or 4 observed more frequently in this group. Chronic HBV infection was the predominant risk factor for HCC in this study (351/602, 58.3%) but was most prevalent in public sector patients (62.1% vs. 17.6%, *p* < 0.00001). No private sector patients were HIV‐infected, whereas 13.8% of public sector patients had HIV (*p* = 0.0014).

**TABLE 1:** Characteristics of patients with hepatocellular carcinoma treated in public and private.

Patient characteristics	Public (*n* = 551)	Private (*n* = 51)	*p*‐value
Age (years)	49.6 ± 14.8	59.7 ± 14.3	< 0.00001
Sex			
Male	408 (74.0%)	38 (74.5%)	0.942
Female	143 (26.0%)	13 (25.5%)	0.942
WHO/ECOG PS			
0	36 (6.5%)	6 (11.8%)	0.161
1	206 (37.4%)	24 (47.1%)	0.174
2	151 (27.4%)	4 (7.8%)	0.0013
3	116 (21.1%)	4 (7.8%)	0.0264
4	28 (5.1%)	0 (0.0%)	0.157
Body mass index (kg/m^2^)	23.6 ± 5.6	27.6 ± 4.7	0.00857
Comorbidities			
Diabetes mellitus	62 (11.3%)	13 (25.5%)	0.00322
Hypertension	122 (22.1%)	10 (19.6%)	0.676
Ischemic heart disease	12 (2.2%)	3 (5.9%)	0.126
Human immune deficiency virus	76 (13.8%)	0 (0.0%)	0.0014
Hepatitis B infection	342	9	< 0.00001
Hepatitis C infection	31 (5.6%)	2 (3.9%)	1
Bilharzia	13 (2.4%)	1 (2.0%)	1
Smoking	162 (29.4%)	11 (21.6%)	0.232
Alcohol intake (any)	171 (31.0%)	16 (31.4%)	0.968
Duration of symptoms (days)	89.3 ± 117.4	51.5 ± 85.1	0.0642
Symptoms			
Pain	395 (71.7%)	22 (43.1%)	0.000024
Weight loss	322 (58.4%)	12 (23.5%)	< 0.00001
Anorexia	136 (24.7%)	5 (9.8%)	0.0174
Nausea	103 (18.7%)	5 (9.8%)	0.113
Vomiting	109 (19.8%)	5 (9.8%)	0.0819
Variceal bleed	31 (5.6%)	2 (3.9%)	1
Fever	23 (4.2%)	1 (2.0%)	0.712
Signs			
Jaundice	86 (15.6%)	1 (2.0%)	0.0055
Ascites	165 (29.9%)	7 (13.7%)	0.0142
Palpable mass	182 (33.0%)	13 (25.5%)	0.267
Hepatomegaly	268 (48.6%)	3 (5.9%)	< 0.00001
Encephalopathy	22 (4.0%)	1 (2.0%)	0.711
Hemoglobin (g/dL)	11.7 ± 2.5	12.4 ± 2.1	0.044
Platelet count (×10^9^/L)	314.6 ± 414.0	212.3 ± 127.2	0.096
International normalized ratio	1.3 ± 0.4	1.2 ± 0.3	0.01
AST (U/L)	194.4 ± 454.8	110.9 ± 168.5	0.161
ALT (U/L)	80.2 ± 110.4	97.1 ± 169.5	0.249
GGT (U/L)	395.0 ± 378.0	202.3 ± 238.1	0.00183
ALP (U/L)	327.1 ± 338.5	157.5 ± 139.1	0.00302
Total bilirubin (μmol/L)	43.5 ± 68.7	26.0 ± 28.0	0.0802
Creatinine (μmol/L)	80.1 ± 51.4	75.6 ± 25.0	0.306
Sodium (mmol/L)	135.5 ± 4.6	137.9 ± 4.6	0.00291
Albumin (g/L)	33.4 ± 8.0	33.6 ± 7.3	0.467
Hepatitis B viral load (IU/mL)	7369548.3 ± 29316463.5	1043319.3 ± 1976274.8	0.333
Child–Pugh grade			
A	253 (45.9%)	23 (45.0%)	0.911
B	198 (35.9%)	8 (15.7%)	0.00355
C	91 (16.5%)	3 (5.9%)	0.180
MELD‐Na score	14.15 ± 6.5	11.27 ± 5.23	0.370
Barcelona clinic liver cancer stage			
0	2 (0.4%)	1 (2.0%)	0.234
A	26 (4.7%)	6 (11.8%)	0.0312
B	31 (5.6%)	6 (11.8%)	0.0808
C	309 (56.1%)	15 (29.4%)	0.000257
D	169 (30.7%)	4 (7.8%)	0.0003
Treatment			
Ablation	14 (2.5%)	5 (9.8%)	0.00453
Resection	43 (7.8%)	26 (51%)	< 0.00001
Liver transplantation	1 (0.2%)	5 (9.8%)	< 0.05
Transarterial therapies	116 (21.1%)	26 (51.0%)	< 0.00001
Sorafenib	46 (8.3%)	1 (2.0%)	0.166
Adriamycin	3 (0.5%)	0 (0.0%)	1
Best supportive care	384 (69.7%)	8 (15.7%)	< 0.00001

Abbreviations: ALP = alkaline phosphatase, ALT = alanine transaminase, AST = aspartate aminotransferase, ECOG = Eastern Cooperative Oncology Group, GGT = gamma‐glutamyltransferase, MELD‐Na = model for end‐stage liver disease‐sodium, PS = performance status, WHO = World Health Organization.

Symptom duration was similar in both groups, but markers of advanced disease predominated in public sector patients. Pain (71.7% vs. 43.1%, *p* = 0.000024), weight loss (58.4% vs. 23.5%, *p* < 0.00001), jaundice (15.6% vs. 2.0%, *p* = 0.0055), ascites (29.9% vs. 13.7%, *p* = 0.0142), and hepatomegaly (48.6% vs. 5.9%, *p* < 0.00001) occurred more often in public sector patients. Public sector patients were significantly more likely to present with advanced (BCLC‐C) (56.1% vs. 29.4%, *p* = 0.000257) or terminal (BCLC‐D) (30.7% vs. 7.8%, *p* = 0.0003) HCC. Moreover, these patients had more multifocal HCC (59.2% vs. 15.7%, *p* < 0.00001), portal vein tumor thrombosis (PVTT) (44.6% vs. 5.9%, *p* < 0.00001), hepatic vein tumor thrombosis (HVTT) (16.5% vs. 0%, *p* = 0.0003) and pulmonary metastases (16.1% vs. 3.9%, *p* = 0.0143) (Table 2). Private sector patients were treated more frequently with ablation (9.8% vs. 2.5%, *p* = 0.00453), liver resection (51.0% vs. 7.8%, *p* = 0.00001), and liver transplantation (9.8% vs. 0.1%, *p* < 0.05), whereas BSC was the predominant modality of care in 69.7% of public sector patients. A significantly greater proportion of private patients were treated with transarterial therapies, 51.0% versus 21.0% (*p* < 0.00001).

**TABLE 2:** Tumor characteristics in public and private patients.

Tumor characteristics	Public (*n* = 551)	Private (*n* = 51)	*p*‐value
Serum alpha‐fetoprotein (μg/L)	105763.2 ± 307996.7	8723.30 ± 33535.9	0.137
Cirrhotic	262 (47.5%)	21 (41.2%)	0.383
Noncirrhotic	229 (41.6%)	15 (29.4%)	0.909
Number of lesions			
1	189 (34.3%)	16 (31.4%)	0.673
≥ 2	326 (59.2%)	8 (15.7%)	< 0.00001
Vascular invasion			
Any PVTT	246 (44.6%)	3 (5.9%)	< 0.00001
Any HVTT	91 (16.5%)	0 (0.0%)	0.0003
Extra‐hepatic metastases			
Lung	89 (16.2%)	2 (3.9%)	0.0143
Bone	22 (4.0%)	0 (0.0%)	0.244
Lymph nodes	15 (2.7%)	0 (0.0%)	0.629
Brain	1 (0.2%)	0 (0.0%)	1
Spine	16 (2.9%)	0 (0.0%)	0.384

Abbreviations: HVTT = hepatic vein tumor thrombosis, PVTT = portal vein tumor thrombosis.

**FIGURE 1:**
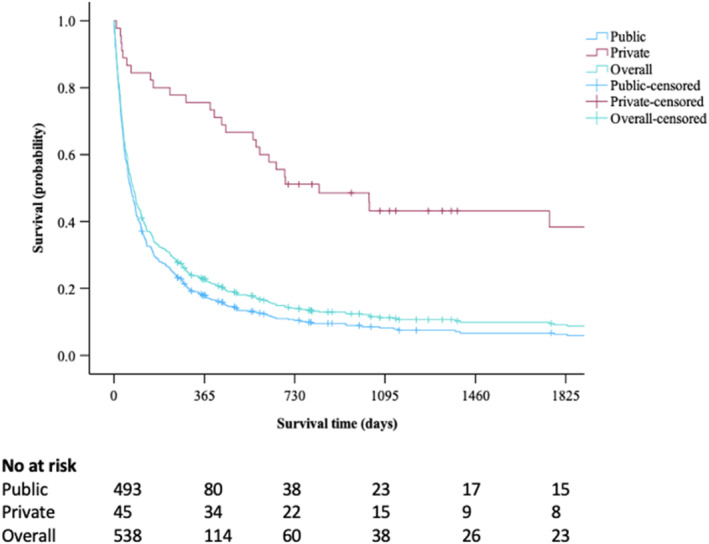
*Survival of entire cohort (78[IQR: 26–288.7]), patients treated in public sector (68[IQR: 25–232]) and patients treated in private sector (703 [IQR: 388–1327]), *p* < 0.001 *Survival expressed as median (days).

The median survival in the entire cohort of 602 patients was 78 [IQR: 26–288.7] days. Overall, survival was significantly lower in patients treated in the public sector, 68 [IQR: 25–232] versus 703 [IQR: 388–1327] days (*p* < 0.001) (Figure 1). Patients treated with curative‐intended therapies had similar outcomes irrespective of where they received treatment. In public and private sector patients, the median survival with ablation and liver resection was 655 [IQR: 372–1002] versus 800 [IQR: 693–828] days (*p* = 0.932) and 563 [IQR: 267–2154.5] versus 929.5 [IQR: 447–1462] days (*p* = 0.976), respectively. A survival comparison was not performed in patients who underwent liver transplantation because this was a small subgroup comprising only seven patients. Patients treated with transarterial therapies in the private sector had better survival, 1031.5 [IQR: 130.5–746.2] versus 242 [IQR: 100.2–489.2] days (*p* = 0.001). In the BSC group, patients treated in the public and private sectors had similar survival, 39 [IQR: 16–95] versus 29.5 [IQR: 25.7–46] days (*p* = 0.995) (Supporting Information Figures S1–S4).

## 4 Discussion

We compared the epidemiology, treatment, and outcomes of HCC in a cohort of 602 patients treated in either a tertiary public or private academic sector hospital, both affiliated with the University of Cape Town. Men comprised three quarters of the patients in the study, and more than half of the patients had positive HBV serology. These features are in keeping with the epidemiology of HCC in SSA [20, 21, 22]. Clinical (ECOG PS 3 and 4, low BMI, weight loss, pain, jaundice, and ascites) and radiological (pulmonary metastases, PVTT, and HVTT) markers of advanced disease were seen more frequently in public sector patients. On the other hand, private sector patients had a disease profile similar to HICs. They were older, had less HBV infection, and had better preserved liver function at presentation as evidenced by lower CTP scores and less BCLC‐C or ‐D disease.

Overall, survival was significantly poorer in the public compared to the private sector patients. This was, however, not due to worse outcomes according to treatment modality in the two groups but rather to the much higher proportion of patients with advanced or terminal disease in the public sector cohort. Given these observations, as would be anticipated, a higher proportion of private patients received curative‐intended therapies. There were no differences in the outcomes of liver resection and ablation between public and private sector patients. Equally, the median survival in patients receiving BSC was poor in both groups. In the public sector, in addition to liver resection, ablation, transarterial therapies (bland transarterial embolization and transarterial chemoembolization), and liver transplantation were gradually offered to public patients as these therapies became available and are now the standard of care. Sorafenib was available as part of a donation program to a limited number of patients at GSH. Currently, sorafenib, novel biological agents such as *atezolizumab and bevacizumab,* transarterial radioembolisation, and stereotactic body radiation therapy are not available in the public sector in South Africa, despite most HCC patients presenting with advanced disease [20, 21, 22].

In spite of the rising cancer burden and the coexistence of usually underfunded public and inaccessible private healthcare systems in many countries throughout SSA, data on disparities in cancer care are limited [5, 12, 22, 23, 24, 25]. In South African cohorts, it has been shown that public sector patients with colorectal cancer and breast cancer have more advanced disease and are less likely to get curative‐intended therapies compared to patients in the private sector [11, 12, 24]. Most data exploring healthcare disparities in the treatment of HCC originate from the United States [23, 24, 25, 26, 27]. In a cohort of 19,059 patients with early‐stage HCC, Gholami et al. concluded that public insurance, lower neighborhood socioeconomic status, rural residence, and care at non‐National Cancer Institute facilities were associated with not receiving treatment and lower survival [23]. An analysis of 43,859 patients from the National Cancer Database found that patients with private insurance, higher income, better education, and treatment at an academic center were more likely to receive surgery for HCC. Private insurance and treatment at an academic hospital were also associated with better survival [24]. Finally, waiting list outcomes for liver transplantation are worse in patients with public insurance compared to those with private insurance [25].

There are some noteworthy studies from Brazil, Russia, India, and China, countries that to some extent have similar healthcare systems and economies to South Africa where HCC patients do not have access to standard therapies advocated by guidelines from HICs [28, 29, 30, 31, 32]. In two Chinese studies examining HCC outcomes, underinsured patients had inferior survival outcomes after liver resection and poorer survival when treated in public facilities [28, 29]. In Brazil, socioeconomic disparities have been directly linked to poorer HCC outcomes and less access to screening programs [30, 31].

In this study, for the first time in SSA, we examined and compared the outcomes of HCC in public and private sector patients. Hepatitis B virus‐related HCC was predominant in the public cohort, where younger patients presented with a higher tumor burden as well as more extrahepatic disease and macrovascular invasion. Many have attributed this SSA disease profile of HCC to the hepatocarcinogenic potential of HBV [33, 34, 35, 36, 37]. Moreover, the lack of public health initiatives, such as the widespread availability of HBV testing, HCC screening in at‐risk populations, and the prevention of mother‐to‐child transmission programs for HBV, has contributed to the rapidly growing HCC pandemic [37, 38, 39].

Over the last few years, a National Health Insurance program aimed at amalgamating the public and private healthcare sectors to ensure universal healthcare coverage was formally proposed in South Africa. Initially proposed as a bill, the National Health Insurance was signed into law in May 2024 [9, 12]. In this novel system, public patients will have state‐funded medical coverage, which will allow them to access facilities in the private sector. However, this program has not been implemented yet and will likely undergo significant challenges in its early phases. Therefore, an immediate impact on HCC care and its outcomes cannot be envisaged in the short term. The findings of this study imply that patients with complex malignancies such as HCC can be treated in public healthcare facilities with outcomes equivalent to the private sector. Although the National Health Insurance Act aims to provide universal healthcare coverage to all South Africans by making use of private healthcare facilities, relevant stakeholders should also look into public investment and capacity building in public hospitals. Such an exercise is more sustainable in the long term and will further improve staff shortages in public hospitals as well as expand and upgrade the infrastructure at many of these institutions. The overall poorer outcomes of public patients are invariably due to the proportion of patients presenting with advanced disease where curative‐intended or life‐prolonging interventions are not feasible. These findings are in line with the results of a systematic review of 39 studies that included 3989 patients from SSA, which showed that BSC was the sole modality of care in 84% of the patients [21].

The findings of this study reiterate the need for stakeholders to focus on preventative health measures such as HBV and HCC screening in at‐risk populations and rejuvenation of infrastructure at public healthcare facilities. Education for the general public and healthcare workers is also critical in this endeavor. Improved access to standardized therapies and palliative care services for patients with HCC should be prioritized. Furthermore, screening of pregnant women with widespread HBsAg testing and the administration of prophylactic tenofovir in the second trimester of highly viremic women (HBV DNA > 200,000 IU/mL) are key preventative measures in limiting the HBV pandemic that drives HCC in SSA. Reemphasizing the integration of the HBV birth‐dose vaccination at all hospitals nationally is a key component of prevention of mother‐to‐child transmission (PMTCT) strategies. A diagnosis of chronic HBV infection at an early age remains associated with an increased risk of cirrhosis and HCC, and thus PMTCT is a sustainable program that can curb the high prevalence of HCC in South Africa and the rest of SSA [39, 40, 41, 42].

## 5 Study Limitations

We acknowledge that social determinants of health such as physical and social environment, public safety, transportation, education, employment, housing, and income were not sought and analyzed for the patients in this study. However, all our private sector patients had medical insurance which is itself a surrogate marker for social determinants of health. In South Africa, it is well recognized that patients with private medical insurance are more likely to be formally employed, come from wealthier households and neighborhoods, have higher levels of education, and have better family support [43, 44, 45].

## 6 Conclusion

In this study, we showed that HCC patients treated in the public sector in South Africa had poorer survival than their private counterparts because of presentation with more advanced disease. Chronic HBV infection remains the main etiology of HCC in our population. Patients treated with curative‐intended therapies had similar survival outcomes irrespective of where they were treated. The development of public awareness and screening programs for at‐risk populations and improvement in infrastructure at state hospitals are key to improving the overall survival of HCC in South Africa.

## Author Contributions

**Sanju Sobnach:** conceptualization, data curation, formal analysis, funding acquisition, investigation, methodology, project administration, resources, validation, visualization, writing – original draft, writing – review and editing. **Muhammad Emmamally:** data curation, formal analysis, investigation, methodology, project administration, resources, software, validation, visualization, writing – original draft, writing – review and editing. **Keith Venter:** data curation, formal analysis, investigation, methodology, project administration, resources, software, validation, writing – original draft, writing – review and editing. **Jake Krige:** methodology, writing – original draft, writing – review and editing. **Marc Bernon:** data curation, methodology, writing – original draft, writing – review and editing. **Christo Kloppers:** data curation, methodology, writing – original draft, writing – review and editing. **Mark W. Sonderup:** data curation, methodology, writing – original draft, writing – review and editing. **C. Wendy Spearman:** data curation, methodology, writing – original draft, writing – review and editing. **Rufaida Khan:** data curation, formal analysis, investigation, methodology, project administration, resources, software, writing – original draft. **Urda Kotze:** data curation, formal analysis, investigation, methodology, project administration, resources, software, writing – original draft. **Eduard Jonas:** conceptualization, data curation, formal analysis, funding acquisition, investigation, methodology, project administration, supervision, validation, visualization, writing – original draft, writing – review and editing.

## Conflicts of Interest

The authors declare no conflicts of interest.

## Supporting information

Supplementary Material

## Data Availability

The datasets generated during and/or analysed during the current study are available from the corresponding author on reasonable request.
