# Supplementary material for: Healthcare Disparities in the Treatment and Outcomes of Hepatocellular Carcinoma in South Africa
Source: World J Surg. 2025 Mar 18;49(5):1290–7. doi: 10.1002/wjs.12559 (PMC12058446; doi:10.1002/wjs.12559)
Supplement: Supplementary file 1 — Supplementary Material [file WJS-49-1290-s001.docx]

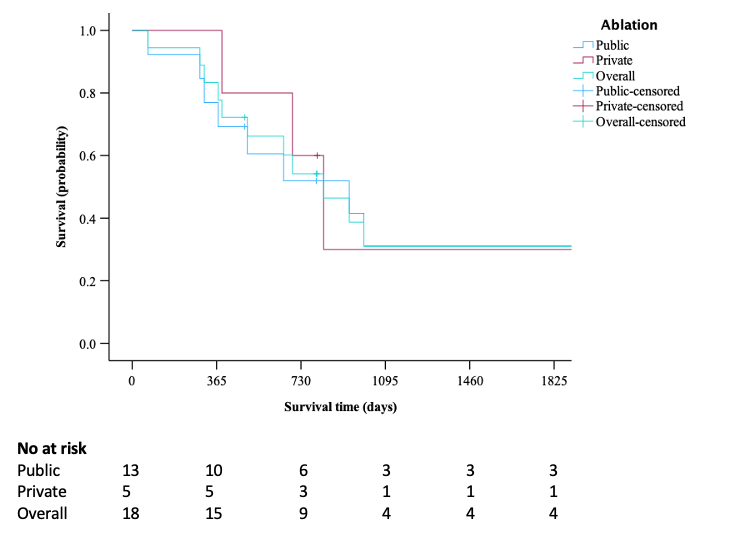


Supplementary figure 1. *Survival outcomes of ablation in entire cohort (744.5[IQR: 412.7-986.2]), patients treated in public (655[IQR: 372-1002]) and patients treated in private (800 [IQR:693-828]), p=0.932

*Survival expressed as median (days)


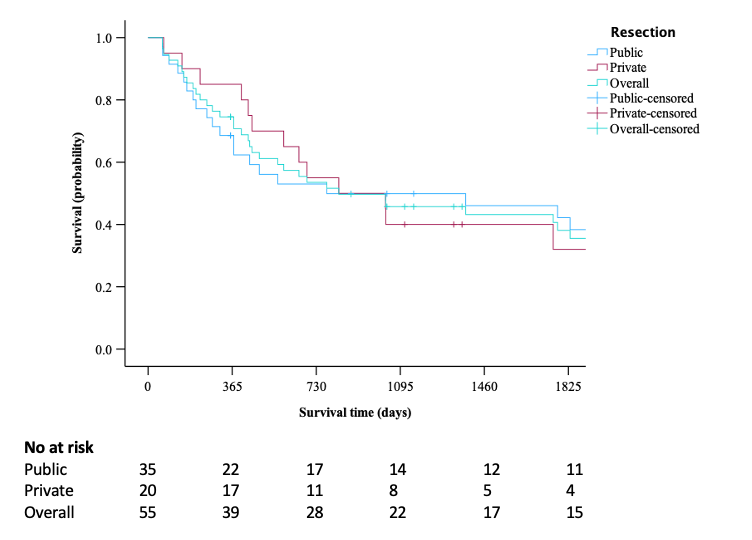


Supplementary figure 2. *Survival outcomes of liver resection in entire cohort (776[IQR: 335-1921]), patients treated in public (563[IQR: 267-2154.5]) and patients treated in private (929.5[IQR:447-1462]), p=0.976

*Survival expressed as median (days)


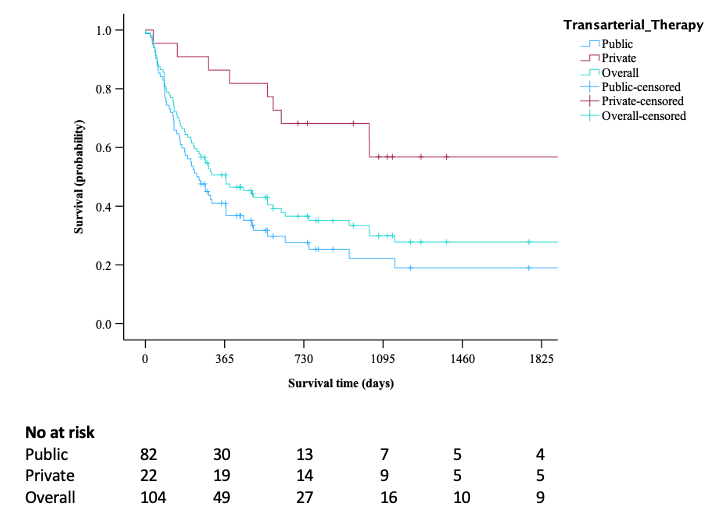


Supplementary figure 3. *Survival outcomes of transarterial therapy in entire cohort (296[IQR: 130.5-746.2]), patients treated in public (242[IQR: 100.2-489.2]) and patients treated in private (1031.5 [IQR:598.2-1358.5]), p=0.001

*Survival expressed as median (days)


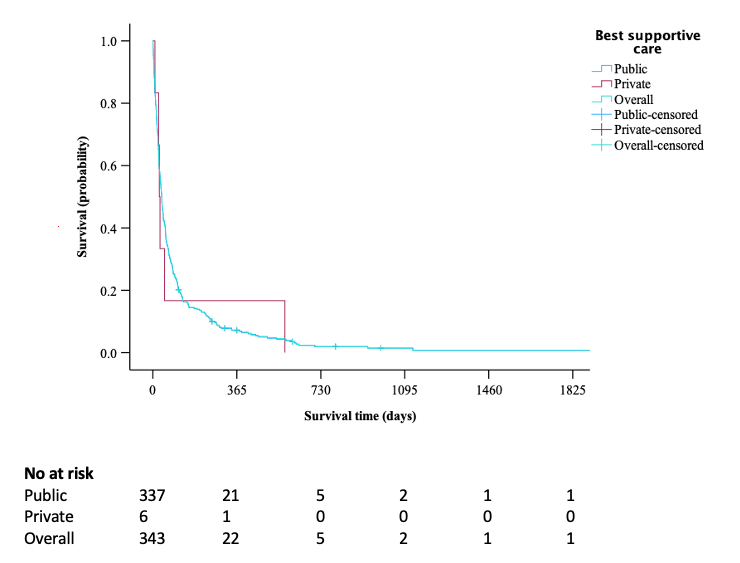


Supplementary figure 4. *Survival outcomes of best supportive care in entire cohort (38[IQR: 16.5-92]), patients treated in public (39[IQR: 16-95]) and patients treated in private (29.5 [IQR:25.7-46]), p=0.995

*Survival expressed as median (days)
